# Body percussion and the biomechanical calibration of musical meter: the somatic externalization of meter hypothesis

**DOI:** 10.3389/fpsyg.2026.1905062

**Published:** 2026-09-09

**Authors:** Francisco Javier Romero-Naranjo

**Affiliations:** Department of Innovation and Educational Training, University of Alicante, Alicante, Spain

**Keywords:** beat perception, biomechanics, body percussion, embodied music cognition, internal models, musical meter, preferred tempo, sensorimotor synchronization

## Abstract

Musical meter takes specific forms, a preferred tempo and a small set of stable subdivisions, that existing accounts do not fully explain. I propose the *Somatic Externalization of Meter* (SEM) hypothesis: that these parameters are calibrated to the oscillatory dynamics of the moving body, so meter inherits the characteristic periods of the limbs. The proposal comprises a well-supported causal claim that bodily and vestibular state shape how an ambiguous rhythm is heard, a central mechanistic claim that beat perception is parameterized by effector biomechanics, and a conjectural claim about manufactured percussion. Body percussion is the diagnostic case, in which effector and instrument coincide, giving a zero-offset baseline against which instrumental displacement is measured. I recast the mechanistic claim as a measurable quantity, *biomechanical transparency*, which admits a strict null, and set out eight falsifiable predictions. Two existing findings run against the hypothesis and are reported.

## 1 Introduction

### 1.1 The puzzle: why does meter take the form it takes?

When people listen to music, they do not merely register a sequence of sounds. They feel a pulse: a regular, recurring beat they can clap, tap, or step to, and that lets them predict when the next event will fall. This felt pulse is remarkably consistent in rate: across a wide range of styles and cultures, the tempo listeners find most compelling clusters within a narrow band near two beats per second, an inter-onset interval of roughly 500 to 600 milliseconds (van Noorden and Moelants, 1999; Moelants, 2002). Faster and slower music exists, but this is the region where synchronization is easiest and a pulse most readily felt.

The felt pulse is also structured. Beats are not all equal: some feel strong and others weak, and the recurring pattern of strong and weak beats is what gives music its sense of being in a march, a waltz, or a more complex grouping. Here too the available forms are restricted. The subdivisions that feel stable are a small set of simple integer ratios, with duple (two-beat) and triple (three-beat) groupings dominating, and the binary case being the widespread default (Snyder et al., 2024).

This regularity is the puzzle the present article addresses. Existing frameworks explain a great deal about *that* meter is periodic and predictive, and about the neural and attentional machinery that tracks it. What they do not explain is *why* meter has the particular periods it has, rather than any others, and why those periods coincide so closely with the natural rhythms of the moving human body. Why two events per second, and not four or one? Why does the binary grouping default match the alternating structure of walking? These are questions about the *values* of the parameters, and the established accounts, for reasons set out in Section 2, leave those values open.

### 1.2 Terms: beat and meter

Because this article addresses readers from beyond the rhythm-perception field, and because researchers do not always use these terms identically, I fix their sense here before building on them.

*Beat* refers to the regular, periodic pulse listeners perceive in music and can synchronize movement to. It is a percept, not simply a property of the signal: a beat can be felt at a rate the sound does not physically mark, and can persist through silences. The rate of the beat is its tempo.

*Meter* refers to the hierarchical organization of beats into recurring patterns of stronger and weaker events, together with the nested subdivisions of the beat into faster levels. Duple meter groups beats in twos, triple meter in threes, and each beat may be divided further. Meter is thus a structured framework of periodicities, of which the beat is one level. Like the beat it is partly perceptual: the same acoustic sequence can support more than one metrical reading, and which reading a listener adopts can depend on factors other than the sound, a fact Section 3 shows to be central here.

Two points follow and matter later. First, an isochronous train of identical tones, such as a metronome, marks a periodic pulse but does not by itself impose a meter, since it supplies no accent structure; meter is more readily studied with rhythms that carry or invite a metrical interpretation. Second, a rhythm can be metrically *ambiguous*, consistent with more than one reading. Ambiguous rhythms are the key experimental tool of Section 3, because they let an experimenter hold the sound constant while varying what the listener does with it.

### 1.3 The proposal in outline

This article advances the *Somatic Externalization of Meter* (SEM) hypothesis. Its core idea is simple to state: the parameters of meter, its preferred tempo and its stable subdivisions, are calibrated to the oscillatory dynamics of the human body’s moving parts, so that meter inherits the characteristic periods of the limbs. On this view, the reason the felt pulse sits near two per second is that this is close to the natural rhythm of the swinging human limb, and the reason the binary grouping is the default is the alternating, left–right structure of the moving body.

The hypothesis is built from three claims, which I distinguish at the outset because they differ sharply in how well they are supported, and because keeping them apart is essential to judging the proposal fairly.

*The causal claim (well supported).* Bodily movement and vestibular state can determine how an ambiguous rhythm is heard. This claim is not original to SEM; it rests on an established experimental literature, reviewed in Section 3.

*The mechanistic claim (the central concern, and the most vulnerable).* The predictive model underlying beat perception is parameterized by the biomechanics of the effector, the moving limb, so that the model’s temporal predictions carry the limb’s mass, length, and natural frequency. This is the claim the article exists to defend and to make testable, and it is developed in Section 4.

*The ontological claim (a conjecture).* Manufactured percussion, the drum and the stick, is a technology that externalizes a bodily timing capacity without replacing it. This is the most speculative claim, developed in Section 5, and it earns its place only by generating testable predictions about body percussion.

### 1.4 The research questions

The article is organized around three questions, one per claim, stated plainly here so the reader can hold them in view.

*Q1 (causal).* Can the state of the moving body determine which meter a listener hears, when the acoustic input is held constant?

*Q2 (mechanistic).* If beat perception recruits the motor system, how far down the motor hierarchy does the recruitment reach, and in particular does it reach the level at which the physical mechanics of the limb are represented?

*Q3 (ontological).* What should we observe in body percussion, the one percussive practice in which no external implement mediates between limb and sound, if meter is indeed calibrated to the body?

Q2 is the pivot of the article, posed as a question of degree rather than a yes-or-no. It is already consensus that beat perception recruits the motor system (Section 4.1); what SEM turns on is whether that recruitment reaches the level at which the limb’s mechanics are represented, or stops at a more abstract level encoding only timing. Section 4.3 develops a measurable quantity, *biomechanical transparency*, to pose this precisely, and shows that it admits a strict null: at its lower limit the body’s mechanics are invisible to meter and the mechanistic claim is false.

### 1.5 What this article does and does not do

This is a Hypothesis and Theory article. It reports no new data; its aim is to specify a position precise enough to be refuted, and to set out the experiments that would refute it. I have tried throughout to report the evidence without selection. Two findings in the literature run against the hypothesis, and rather than leaving them to be discovered I introduce them where they arise: a null anthropometric result in the study most cited for the coincidence of musical and locomotor tempo (Section 4.2), and an absence of within-limb differentiation in the only kinematic study of body percussion (Section 5.3). Neither is fatal, but neither is hidden.

That the article’s central predictions are new is not a rhetorical claim. A bibliometric survey of 245 body-percussion documents indexed between 2001 and 2022 finds the field overwhelmingly pedagogical and clinical (Arnau-Mollá and Romero-Naranjo, 2024): body percussion has been studied as an educational tool, not as a window onto beat perception. The gap runs both ways, as rhythm cognition has correspondingly ignored corporeal percussion as an object of theoretical interest (Fitch, 2016).

### 1.6 Organization

Section 2 situates SEM among the frameworks with which it might be confused and states where it departs. Section 3 develops the causal claim (Q1); Section 4 the mechanistic claim, posing Q2 as a question of biomechanical transparency; Section 5 the conjectural claim, body percussion, and Q3. Section 6 sets out eight falsifiable predictions, each paired with the rival it discriminates against, and Sections 7 and 8 discuss and conclude.

## 2 Situating the hypothesis among existing frameworks

Before developing SEM, I place it against the accounts of rhythm perception it must be distinguished from. The point of this section is not to survey the field but to establish two things: what SEM shares with existing frameworks, and the single respect in which it goes beyond them. Readers already familiar with this literature may move quickly; readers new to it will find here the background the later arguments assume.

### 2.1 The established accounts

Five frameworks dominate current thinking about beat and meter. I state each in a sentence, with the feature relevant to SEM.

*Neural resonance theory* holds that beat and meter arise when networks of neural oscillators entrain to the acoustic signal, supported by frequency-tagging studies in which the electroencephalogram shows sustained responses tuned to metrical frequencies, including ones imagined rather than physically present (Large and Snyder, 2009; Nozaradan et al., 2011; Nozaradan et al., 2012). Its most recent synthesis adds Hebbian learning at the oscillator connections, so that natural frequencies and coupling strengths are progressively tuned by the temporal structure the network is repeatedly driven with Harding et al. (2025). This matters for SEM, because oscillator parameters are then not fixed constants but quantities shaped by experience, which raises the question of which experience does the shaping.

*Dynamic attending theory* models attention itself as an oscillatory process that entrains to environmental rhythm, so that attentional energy peaks in time with expected events (Jones and Boltz, 1989; Large and Jones, 1999).

*Predictive coding and active inference* treat meter as a hierarchical generative model whose prediction errors drive perception; in the active-inference formulation, perceiving and moving are two ways of minimizing the same prediction error (Vuust and Witek, 2014; Koelsch et al., 2019).

*Active sensing* shows empirically that motor engagement structures the temporal precision of auditory attention, so that the motor system helps decide when the auditory system listens most acutely (Morillon et al., 2014; Morillon et al., 2015).

*Action Simulation for Auditory Prediction* (ASAP) proposes that beat perception depends on a covert simulation of periodic movement in motor-planning regions of the brain, and that this simulation supplies the predictive signal the auditory system uses to anticipate the next beat (Patel and Iversen, 2014; Cannon and Patel, 2021). ASAP is the framework SEM is closest to, and the one it must most carefully be distinguished from.

Alongside these, the embodied music cognition tradition has long argued that musical structure is grounded in sensorimotor engagement rather than in abstract symbol manipulation (Leman, 2007; Leman and Maes, 2015; Maes, 2016). Music-induced movement period-locks to metrical levels, with faster limb movements tracking faster subdivisions (Burger et al., 2014), and it has been argued directly that dance and the moving body have been wrongly separated from the study of meter and groove (Fitch, 2016).

Independently of this recent work, and anticipating much of it, Todd and colleagues advanced a sensory-motor theory of rhythm in which beat induction is a form of sensory-guided action, the felt sense of motion from sound is mediated by the vestibular system, and the motor representation of the body carries certain natural frequencies to which rhythm perception is referred (Todd and Lee, 2015). SEM is, in an important sense, a descendant of this line of thinking, and I return to it in Section 3.

### 2.2 What SEM shares with these accounts, and where it departs

I begin with what SEM concedes, because the sharpest objection is that it says nothing new. The motor system’s involvement in beat perception is consensus, not an SEM claim: the reciprocal tuning of perceptual and motor dynamics over development is implicit in ASAP, explicit in neural resonance theory through Hebbian oscillator learning, and a design principle of active inference. A proponent of any of these might reasonably say that SEM articulates something their framework already permits. I accept this and regard it as a strength: a hypothesis incompatible with all the leading accounts would almost certainly be false.

The departure turns on a distinction that must be made explicit. Each of these frameworks *permits* the parameters of the metrical model to be set by effector biomechanics; none of them *requires* it, and each remains fully coherent if they are not. Neural resonance theory does not specify what fixes oscillator frequencies. Dynamic attending does not specify the period of the attending rhythm. Predictive coding does not specify the temporal grain of the priors. ASAP does not specify at what level of the motor hierarchy the simulation operates. A framework that permits a claim and a hypothesis that asserts it are not equivalent, because only the second can be refuted.

SEM converts permission into commitment, and pays for the commitment in falsifiability. Its distinctive signature can be stated in one sentence: physically altering the biomechanics of an effector, while holding the acoustic input and the listener constant, should shift preferred tempo and subdivision in a predictable direction and by a predictable amount. No substrate-general account predicts this, because a change in the mass of a limb changes nothing in the acoustic input and nothing in a neural oscillator. This is the commitment the rest of the article defends and makes testable.

## 3 The causal claim: can the body determine what is heard?

This section addresses Q1. It develops the best-supported of the three claims, and it is the foundation on which the others rest. If meter were simply read off the acoustic surface, bodily movement could at most accompany a structure already fixed by the sound, and SEM would collapse into the conventional picture it means to displace. The evidence shows otherwise.

### 3.1 Movement and vestibular state determine meter

The central paradigm is elegant. Listeners hear a metrically ambiguous rhythm: a repeating pattern with no acoustic accents, equally consistent with a duple (march-like) or a triple (waltz-like) reading. During exposure, the listener’s body is moved, bounced by an experimenter, on every second beat in one condition and on every third beat in another. The metrical interpretation the listener subsequently adopts follows how the body was moved: those bounced on every second beat later hear the pattern as duple, those bounced on every third beat hear it as triple (Phillips-Silver and Trainor, 2005, 2007). The sound is identical across conditions. What differs is what happened to the body, and that is what changes the percept. This is the answer to Q1: yes, bodily state can determine which meter is heard, with the acoustic input held constant.

Three features make this evidence pointed rather than merely suggestive, and the third requires a distinction that bears directly on the mechanistic claim developed later.

First, the effect appears in seven-month-old infants, before extensive enculturation into any particular metrical system, which indicates that the movement-to-meter mapping is foundational rather than a late cultural overlay (Phillips-Silver and Trainor, 2005). That beat sensitivity is present very early is independently supported by electrophysiological evidence that even newborn infants register the periodicity of a rhythmic sequence and detect omissions of the downbeat (Winkler et al., 2009).

Second, it is the listener’s own bodily state that matters: passively watching another person being moved does not produce the bias, whereas being moved oneself does (Phillips-Silver and Trainor, 2007). This rules out a purely visual or attentional account and implicates the participant’s own sensorimotor and vestibular state.

Third, and requiring the most care, the driving signal is vestibular. Here I must be exact, because the manipulation and its interpretation are easily conflated. In these studies the infant does not move its own body under its own motor commands; it is *moved* by an experimenter who bounces it. What varies across conditions is therefore not the participant’s motor output but the pattern of vestibular and proprioceptive stimulation the passive movement produces. That the critical variable is vestibular, and not motor, is shown directly by two results: head movement specifically drives the effect, and galvanic stimulation of the vestibular nerve shifts auditory metrical interpretation in the complete absence of any movement at all (Phillips-Silver and Trainor, 2008; Trainor et al., 2009). The relevant variable is thus a bodily signal of motion, not the execution of a movement and not its acoustic consequences.

This distinction is not a quibble, and I flag it now because it constrains what the causal claim licenses. The bouncing paradigm is strong evidence that the *vestibular* system is upstream of the felt pulse; it is not, on its own, evidence that the participant’s own *motor* system parameterizes meter, since in these studies the participant’s motor system is not what is manipulated; the case for motor involvement is made separately, on different evidence, in Section 4. Todd and colleagues anticipated exactly this division of labor, assigning the sense of motion from sound to the vestibular system while treating beat induction as sensory-guided action referred to the natural frequencies of the body’s motor representation (Todd and Lee, 2015). SEM inherits both halves of that picture but keeps them distinct, because the evidence for each is distinct.

### 3.2 Synchronization is anticipatory, which is why it reveals the body

The second strand of the causal claim concerns not perception but the coupling of perception to action. When people tap along with a regular auditory pulse, their taps do not follow the tones; they slightly *precede* them, typically by tens of milliseconds. This negative mean asynchrony is one of the most robust findings in the tapping literature (Repp, 2005; Repp and Su, 2013). A tap is therefore not a reaction to the beat but the expression of a prediction about when the beat will fall.

This matters for SEM in a specific way. If synchronization were reactive, the timing of a tap would tell us about auditory processing and nothing about the body. Because it is anticipatory, the tap reveals the state of the *predictive model*, and the predictive model is exactly the object about which SEM makes its central claim. The negative mean asynchrony is thus not a curiosity of tapping but a window onto the parameters of the model that generates the felt beat, the same model whose calibration Section 4 concerns.

Two clarifications are needed, both of which the terminology can obscure. The first concerns the metronome. Much of the synchronization literature uses an isochronous metronome, and it is tempting to treat tapping to it as tapping to a beat within a meter. Strictly, it is not: as noted in Section 1.2, a metronome marks a periodic pulse but supplies no accent structure and so imposes no meter, and a participant may tap each click one-to-one without forming any hierarchical percept. Evidence bearing on *meter* specifically comes instead from paradigms in which listeners tap at designated metrical levels of a genuinely metrical rhythm, or in which the electroencephalographic response is measured at metrical frequencies absent from the stimulus (Nozaradan et al., 2011, 2012; Repp and Su, 2013). Where the distinction matters below, I mean the latter.

The second concerns prediction. The negative mean asynchrony is the clearest *signature* of prediction in synchronization, but not its only route. Even where taps do not lead the tone, maintaining synchrony with a periodic signal requires anticipating upcoming events and correcting against that anticipation rather than reacting to each event as it arrives (Repp and Su, 2013). Synchronization is thus predictive whether or not it shows a negative asynchrony, which matters because the argument of Section 4 depends on its predictive character, not on the sign of the asynchrony.

One further constraint is worth recording for later use: synchronization is most stable within a limited tempo band and degrades sharply outside it (van Noorden and Moelants, 1999; Repp, 2005). Together with the early emergence of beat sensitivity noted in Section 3.1, this indicates a capacity that is not learned from scratch but whose parameters are, on the SEM reading, set by the body it is learned in Honing (2012).

## 4 The mechanistic claim: a predictive model calibrated to the body

Section 3 established that the body is causally upstream of the felt pulse. This section asks *why*, and *how*. Why should the body shape meter, and shape it in the particular way it does? The mechanistic claim proposes an answer: the predictive model that supports beat perception is calibrated to the oscillatory dynamics of the human effector system, so that its temporal predictions carry the mass, length, and natural frequency of the moving limb. This is the claim on which the article stands or falls. I develop it in three steps, moving from what is established to what SEM alone asserts, and I flag at the outset that the crucial step depends on a reading of the evidence that a resonance theorist could resist. Section 6 is designed to test exactly that reading.

### 4.1 Beat perception recruits the motor system

The first step is not controversial. That beat perception engages the motor system, even when the listener does not move, is well established. Functional imaging shows the basal ganglia and supplementary motor area responding more strongly to beat-inducing rhythms than to matched non-inducing ones, during passive listening (Grahn and Brett, 2007). The striatal contribution is specifically predictive, being greatest during beat continuation and least during beat finding, which locates the basal ganglia in beat prediction rather than discovery (Grahn and Rowe, 2013). Parkinson’s patients show impaired discrimination of beat-based rhythms in particular, implicating the basal ganglia causally (Grahn and Brett, 2009), and auditory-motor coupling in music is by now a standard finding (Zatorre et al., 2007; Chen et al., 2008).

ASAP builds on this, proposing that the motor system’s contribution is predictive in a specific way: a covert simulation of periodic movement in motor-planning regions supplies the timing signal the auditory system uses to anticipate beats (Patel and Iversen, 2014; Cannon and Patel, 2021). None of this is SEM’s contribution, and I claim none of it. It is the platform on which SEM’s own step is taken.

### 4.2 From motor prediction to bodily parameters: the coincidence of tempi

The step that is properly SEM’s own is the inference from *the motor system predicts the beat* to *the beat carries the parameters of the moving body*. The reasoning is this: the motor system does not predict time in the abstract; it predicts the timing of movements, and movements are made by physical limbs with mass, inertia, and characteristic oscillatory periods. A prediction about the timing of a limb’s movement is therefore, unavoidably, shaped by that limb’s mechanics.

The most striking supporting evidence is a numerical coincidence. The preferred and most stable region for the human beat clusters near an inter-onset interval of 600 milliseconds, roughly two events per second (van Noorden and Moelants, 1999; Moelants, 2002). Measuring head acceleration over ten hours of unconstrained daily activity in 20 participants, MacDougall and Moore (2005) report a sharply tuned locomotor frequency at 2 Hz. That the most compelling musical pulse and the natural rhythm of the moving body coincide so precisely is, on the SEM reading, no accident (Patel and Iversen, 2014; Repp and Su, 2013), and the binary metrical default has a parallel explanation in the alternating structure of bipedal gait (Larsson et al., 2019).

#### 4.2.1 A finding that cuts against SEM, stated in full

The same study reports something SEM must confront rather than pass over. MacDougall and Moore found no correlation between the 2 Hz locomotor frequency and the height, weight, age, sex, or body mass index of their participants. On the strength of this null result they propose that the tempo reflects a *central* resonant frequency of human movement, mediated by otolith-spinal reflexes, rather than a *peripheral* biomechanical one. Read straightforwardly, this is evidence against the biomechanical parameterization SEM proposes: if limb length fixed the locomotor period, taller people should walk more slowly, and in this dataset they did not.

Three things can be said, and none dissolves the problem. First, walking cadence is not the same measure as preferred musical tempo, and the two need not scale together: in a study designed to detect exactly this relationship, Dahl et al. (2014) find that height and leg length combined best predict preferred *dance* tempo, with no independent effect of sex once height is matched. Second, walking cadence is under strong optimization pressure for metabolic efficiency, which may compress inter-individual variation. Third, and most importantly, the disagreement between these studies is precisely what SEM’s decisive experiment would settle: if biomechanical transparency is zero, the central-resonance reading is correct and SEM’s mechanistic claim fails.

I therefore cite MacDougall and Moore (2005) for the coincidence of tempi, which it firmly establishes, and record openly that its own authors interpret their null anthropometric result against the reading I am proposing.

### 4.3 Q2 made precise: at what level of the motor hierarchy is meter parameterized?

The inference of Section 4.2, from *the motor system predicts the beat* to *the beat carries the parameters of the moving body*, is the load-bearing step of the paper. It is tempting to treat it as obvious, and it is not. This section supplies the argument in the form the evidence actually supports, which is weaker and more interesting than a straight deduction: not a proof that meter is body-parameterized, but a measurable quantity that the existing frameworks leave undetermined and that SEM’s experiments would fix.

#### 4.3.1 Motor prediction can, in principle, represent the limb’s mechanics

The internal-model tradition in motor control begins from the idea that the central nervous system maintains a *forward model*: a neural simulation that takes an efference copy of a motor command and predicts its sensory consequences (Wolpert et al., 1995). The point that matters here is what such a model must be a model *of*. A forward model that predicted the consequences of a command without representing the dynamics of the limb executing it would be useless, because the same command applied to a heavy limb, a loaded limb, or a limb moving in an unfamiliar force field produces entirely different consequences. A forward model in this sense is a model of the *plant*: of a physical system with mass, inertia, viscoelasticity, and characteristic natural frequencies. Its parameters are the plant’s parameters.

The empirical support is direct. When participants reach in a novel force field that alters the arm’s dynamics, they adapt by acquiring an internal model of the new limb dynamics, not by memorizing a new trajectory (Shadmehr and Mussa-Ivaldi, 1994). The cerebellum has been characterized as the substrate of such models (Wolpert et al., 1998), and optimal feedback control shows that the control policy itself is derived from the plant’s dynamics and the cost of moving it (Todorov and Jordan, 2002; Scott, 2004).

This establishes something necessary for SEM but far short of sufficient. Force-field adaptation shows that plant-parameterized predictive models *exist* in the human motor system and are learnable from experience; had they not, SEM would be ruled out immediately, for want of any body-parameterized model for meter to draw on. Their existence makes the hypothesis *possible* without making it *probable*: these results do not show that beat perception is body-parameterized, and do not even bear on it. Whether meter draws on such models, and if so how far, is the separate question the rest of this section addresses.

#### 4.3.2 Why the argument cannot simply be completed

It would now be tempting to finish in one move: ASAP holds that beat perception co-opts the motor system’s predictive apparatus; a predictive model of movement is a model of the plant; therefore beat perception inherits the plant’s parameters. This move does not go through, and the point at which it fails is the most important thing this section has to say.

The motor system is not a single predictive model. It is a hierarchy, and its levels are parameterized differently. A movement can be specified in *task space*, as a goal, a target, a timing, a trajectory in external coordinates. Or it can be specified in *plant space*, as the pattern of joint torques and muscle activations required to realize that trajectory given the mass, inertia, and viscoelasticity of the limb that must execute it. Both are motor representations, but only the second is body-parameterized. The distinction is standard and well evidenced: kinematic planning in task coordinates is routinely dissociated from dynamic specification in intrinsic coordinates, and the transformation between them is precisely what the internal model performs (Wolpert et al., 1995; Todorov and Jordan, 2002; Scott, 2004).

ASAP is a claim about the recruitment of motor *planning* and covert *simulation*. It does not specify which level of the hierarchy is recruited. A simulation operating at the task level would carry temporal structure, predicting *when*, without carrying limb mass or length, because those quantities do not appear in a task-space representation. On such a reading, beat perception could co-opt the motor system entirely and still generate metrical parameters wholly indifferent to effector biomechanics. This is not a marginal possibility; it is a coherent, defensible, and arguably the *default* reading of ASAP, and a reader who holds it has misunderstood nothing.

#### 4.3.3 The question posed as a quantity: biomechanical transparency

The situation is therefore not one in which SEM follows deductively from ASAP plus the internal-model literature. It is one in which the two frameworks jointly generate a question that neither answers: how far down the motor hierarchy does the recruitment reach? Q2 is that question, and it must be posed carefully, because the obvious way of posing it is wrong.

The obvious way is a dichotomy: either the simulation is task-level, in which case SEM is false, or plant-level, in which case SEM is true. I reject this framing on a substantive point of motor architecture: motor control is not cleanly stratified into two discrete, sequential levels. Task-level and plant-level representations interact continuously rather than in a strict pipeline, and the transformation between them is graded rather than switch-like, since in optimal feedback control the policy is computed from task cost and plant dynamics together rather than by translating a fixed task-level plan, so the two levels are coupled throughout the movement (Todorov and Jordan, 2002; Scott, 2004; and on the internal model as the graded transformation between them, Wolpert et al., 1995). A dichotomy would therefore rest on a premise the motor-control literature does not support.

The question is better posed as one of degree. Let the *biomechanical transparency* of the metrical model be the extent to which the parameters of the effector system are visible to it. Transparency is a quantity, not a switch, and it can take any value between two limits.

*At the lower limit, transparency is zero.* The simulation supplying temporal prediction operates wholly in task coordinates: it predicts when, and nothing about limb mass or length enters. Preferred tempo and subdivision are then indifferent to effector mechanics, loading moves nothing, and the coincidence of preferred tempo with the cadence of gait must be explained some other way. At this limit SEM’s mechanistic claim is simply false.

*At the upper limit, transparency is complete.* The simulation descends fully to the level at which the mechanical plant is represented, and the temporal predictions carry the plant’s parameters in full. Preferred tempo and subdivision then track effector mechanics exactly, and are displaced precisely by the mass of an implement. At this limit SEM’s mechanistic claim holds in its strongest form.

Between the limits lies the interesting territory, where I expect the answer to fall. The metrical model may be sensitive to gross effector identity, whether one is clapping, tapping, or stamping, without tracking fine variations in loading within a single effector; it may track limb length but not limb mass; it may be transparent to mechanics developmentally but opaque acutely. Each is a distinct intermediate value of transparency, and each yields a distinct, detectable pattern across the predictions of Section 6.

What makes the hypothesis testable despite the absence of a clean dichotomy is that transparency has a measurable signature whose lower limit is a strict null. Adding mass to an effector changes the plant while leaving the task specification untouched, so a model with zero transparency cannot see the manipulation and must predict invariance. Any measurable displacement of preferred tempo therefore places transparency above zero, and its magnitude, relative to what the altered mechanics predict, estimates how far above. The experiment does not merely choose between hypotheses; it measures a parameter that can come out zero.

This is the central move of the article. The science of rhythm has established that beat perception recruits the motor system, but not how far down the hierarchy the recruitment reaches, and the metrical consequences depend on the answer. A null result is then not a disappointment but a measurement: invariance under loading would show transparency is near zero and that recruitment stops at the task level, itself a substantive finding that would constrain ASAP, neural resonance theory, and predictive coding alike, none of which currently commits on the point.

#### 4.3.4 Has the decisive manipulation been done? An honest answer

For meter specifically, it has not. No published study, to my knowledge, has altered the mass, inertia, or length of an effector while holding the acoustic stimulus and the listener constant, then measured the shift in preferred tempo or subdivision. This is a genuinely novel prediction, not a redescription of an existing result, and where the hypothesis is most vulnerable. What exists is adjacent evidence that makes it plausible rather than gratuitous.

Across bodies, height and leg length together best predict preferred dance tempo (Dahl et al., 2014); this is variation across individuals rather than the within-body causal shift SEM predicts, and I do not overstate it. Finger-tapping and whispering show distinct optimal rates, which the authors read as evidence for partially distinct rhythmic-timing mechanisms (Barchet et al., 2024). Movement both biases duration estimates and makes them more precise, on a Bayesian account in which movement affords the most precise representation of time (de Kock et al., 2021; Robbe, 2023). Body movement selectively shapes the neural representation of a rhythm, enhancing the response at the movement frequency while the acoustic input is unchanged (Chemin et al., 2014), and a drummer’s kinematics shape a listener’s experience of tempo, carrying timing information the sound alone does not fix (Burger and Wöllner, 2023).

More directly relevant is evidence for a bodily hierarchy of effectors. Using five effectors (left foot, right foot, left hand, right hand, voice), Mårup et al. (2024) report that performing a rhythm against the hierarchy increases tapping errors and recruits regions of top-down correction and prediction error, while performing with it engages regions of automatization. Effectors are thus ranked, and the ranking has neural consequences. SEM’s further claim, that the ranking is mechanical rather than an arbitrary neural ordering, goes beyond what Mårup et al. establish; but that a hierarchy exists, and that violating it costs the system something measurable, is what a body-parameterized account predicts and a substrate-general account does not. Finally, in speech the natural frequency of the orofacial articulators fixes the syllabic rate to which auditory cortex is tuned, structurally SEM’s argument in a domain where it is better evidenced (Poeppel and Assaneo, 2020).

#### 4.3.5 Which motor-control frameworks support a body-parameterized model

Three levels of description converge on the possibility SEM requires. *Internal models* supply the computational rationale: a predictive model of movement must represent plant dynamics or it cannot predict (Wolpert et al., 1995; Wolpert et al., 1998). *Optimal feedback control* supplies the reason the parameters should be stable and characteristic rather than arbitrary: the control policy is optimized for a specific plant and a specific cost, so the resulting movement has a time structure that is a function of limb mechanics (Todorov and Jordan, 2002; Scott, 2004). *Neural resonance theory*, in its Hebbian formulation, supplies the mechanism of tuning: if oscillator frequencies are tuned by the temporal statistics of the signals that repeatedly drive the network, the question becomes which signals drive it from birth, and SEM’s answer is the proprioceptive, vestibular, and efferent signals whose statistics are fixed by the mechanics of the developing body (Harding et al., 2025). If a neural-resonance proponent says that SEM specifies what the Hebbian tuning signal consists in, I agree, and regard that as the correct integration rather than a concession.

The application of internal models to music perception is not new. Maes et al. (2014) explain the reciprocal influence of action on music perception through inverse and forward modeling. SEM is a specification within that framework rather than an alternative: where Maes et al. establish that internal models mediate action-perception coupling in music, SEM asks what fixes the *parameters* of the model so mediated, and answers that the effector’s mechanics do.

#### 4.3.6 The timescale problem: three claims that must not be conflated

The most serious objection to SEM’s decisive prediction is not that the body is irrelevant to meter but that the coupling between neural and bodily dynamics is established over developmental timescales and cannot plausibly be re-tuned by a temporary load within a single session. The objection is well taken, and meeting it requires distinguishing three claims that are easily run together.

*Claim A: developmental calibration (slow, over years).* The metrical model acquires its parameters through the reciprocal tuning of neural and bodily dynamics across development. This is the claim for which neural resonance theory’s Hebbian learning now supplies a mechanism (Harding et al., 2025), and it is compatible with the developmental narrowing of metrical sensitivity (Hannon and Trehub, 2005a, 2005b; Snyder et al., 2024). On this claim SEM is not radical.

*Claim B: chronic biomechanical difference (intermediate, over years of specialization).* Populations whose effector mechanics differ chronically, through anthropometry, through the specialized training of body percussionists or tap dancers, through amputation, or through altered gait, should show correspondingly different metrical parameters. This is testable now, and the anthropometric result of Dahl et al. (2014) is a first indication in its favor.

*Claim C: acute recalibration (fast, minutes to hours).* Temporarily altering effector mechanics should shift metrical parameters within a session. This is the strongest and most vulnerable claim, and it is the one the objection targets.

I now weaken Claim C in the direction the objection requires, which makes it more informative rather than less. SEM does not predict that a wrist weight will re-tune the covert metrical model as decades of embodied experience would. It predicts a *gradient of malleability* ordered by how directly each measure depends on the online state of the effector:

1. Sensorimotor synchronization with the loaded effector should shift most, and shift immediately. Here the objection of circularity must be faced squarely: if altering an effector’s mechanics alters the timing of movements of that effector, the result is close to trivial. I concede this. Loaded-effector synchronization is therefore a *manipulation check*, not a test of the hypothesis, and I do not offer it as evidence for SEM.

2. Preferred tempo and subdivision judgments made while the effector is loaded, but measured perceptually: the participant adjusts a metronome to the most comfortable tempo, or judges which of two subdivisions best fits a rhythm. These are the genuinely diagnostic acute measures, because the dependent variable is a perceptual judgment while the independent variable is peripheral mechanics.

3. Covert metrical interpretation of an ambiguous rhythm, measured without movement (frequency-tagged EEG, mismatch negativity, or attentional paradigms) in a participant whose effector has been loaded but who is not moving. SEM predicts a *small* effect here acutely, and this is where the hypothesis is most at risk. A null at level 3 with a robust effect at level 2 would not falsify SEM; it would locate the coupling in the online state of the motor plan rather than in the perceptual model, which is itself an informative outcome.

4. Transfer to unloaded effectors. SEM predicts no transfer acutely: loading the right arm should not shift the preferred subdivision expressed through the feet. An account on which movement merely raises general arousal or attention predicts transfer. This dissociation is the cleanest available discriminator, and it is why effector-specific transfer (Prediction 2) is offered alongside the loading manipulation.

Stating the gradient this way costs SEM its most dramatic claim and gains it a pattern of results, rather than a single effect, that fails informatively at each level. The objection is thus not deflected but built into the hypothesis.

#### 4.3.7 Scope of the effector system: limbs and the orofacial case

SEM is not restricted to gross limb movement. The mechanism is indifferent to which effector is at issue: any effector with mass, inertia, and a characteristic natural frequency should contribute its parameters to the timing model that simulates it. The orofacial case is the strongest available analog, because the argument SEM makes for meter has already been made, and better evidenced, for speech: the natural frequency of the jaw and orofacial articulators corresponds closely to the syllabic rate at which speech is produced and to which auditory cortex is most sensitive, and that correspondence is explanatory rather than coincidental (Poeppel and Assaneo, 2020). SEM’s claim about limbs and meter is structurally the same claim transposed to a different effector, and it yields Prediction 6.

## 5 The conjectural claim: externalization, culture, and body percussion

This section addresses Q3, and it develops the most speculative of the three claims, which I mark as such throughout. The claim is this: if meter is generated by a body-calibrated predictive model, then manufactured percussion, the drum, the stick, the mallet, can be understood as a *technology that externalizes that model rather than replacing it*. The player’s bodily timing capacity is not offloaded to the instrument; the instrument is driven by it. On its own this is a conceptual reframing, and it would be idle. It earns its place only by generating the concrete predictions set out below, which are not conceptual at all. I first bound the claim against what the cross-cultural evidence forbids (5.1), then state the reasoning that makes body percussion the diagnostic case (5.2), then present the one existing dataset that bears on it (5.3).

### 5.1 Culture, and the limits of the claim

SEM is not a nativist thesis about metrical universals, and the enculturation evidence forbids one. Infants discriminate the complex, non-isochronous meters of Balkan music as readily as the simple meters of Western music, and this culture-general sensitivity narrows with exposure, so that by twelve months infants show the culture-specific pattern of adults (Hannon and Trehub, 2005a, 2005b; Hannon et al., 2012). Metrical competence is learned, and learned early.

Nor is beat-based timing the categorical human innovation it was once taken to be. Macaques synchronize to a subjective beat in real music, spontaneously and in preference to alternative strategies (Rajendran et al., 2025). SEM neither requires nor predicts a sharp human-macaque discontinuity: a graded distribution of beat-based timing across primates is what a hypothesis grounding meter in effector mechanics shared with other primates should expect.

Cross-cultural work on rhythm priors, using iterated reproduction, finds peaks at simple integer ratios in both US participants and members of a native Amazonian society, but the priors of the two groups are distinct, and the authors are explicit that their data, while not precluding a biological constraint favoring integer ratios, suggest that rhythm priors are substantially modulated by experience (Jacoby and McDermott, 2017). I cite the study for the cross-cultural presence of integer-ratio structure, and note that its authors read their own data as favoring an experiential rather than a constitutional explanation.

SEM must be stated carefully to remain compatible with all this. The body does not *determine* meter; it *constrains the space* within which culture builds. The periods and stability zones available to any musical culture are bounded by the mechanics of the human effector system, and cultures differ in which regions of that space they occupy, not in the bounds themselves. This is a claim about the envelope, not its contents.

### 5.2 Why body percussion is the diagnostic case

The reasoning is mechanical. Every percussive act couples an oscillating effector to a sounding surface, and the timing is governed by the dynamics of whatever is actually oscillating. In manufactured percussion, that is not the limb alone but the limb *plus* an implement: a stick with its own mass and length, a mallet whose rotational inertia about the wrist adds to the arm’s own. The effective natural frequency of this compound system is displaced from the limb’s unloaded value, in a direction elementary mechanics specifies: adding mass distally lowers the natural frequency, and more the further from the axis of rotation it sits. This is not conjecture; percussive gesture has been modeled within exactly the internal-model framework of Section 4, with the implement’s mechanics entering as parameters (Bouënard et al., 2012).

Body percussion, the handclaps of flamenco *palmas*, the foot percussion of tap and *zapateado*, *hambone*, gumboot, the Bavarian *Schuhplattler*, the Basque *Esku dantza*, is the unique case in which this displacement is zero. The effector and the instrument are the same object; the hand that strikes the thigh is the hand whose mechanics govern the strike. There is no implement and no added mass, and therefore no offset between the natural frequency of the effector and that of the sounding system. The acoustics of the unmediated case, the handclap, have been characterized in detail (Repp, 1987). This yields two predictions, stated before defending them.

*Zero offset (Prediction 4).* In body percussion, preferred striking rates and the most stable subdivisions should coincide with the natural oscillatory frequencies of the limb segments employed, with no systematic offset. A substrate-general account predicts instead that preferred rates in body percussion should be the same as on any other instrument, because on that view the parameters are neural and the sounding surface is irrelevant.

*Instrumental offset (Prediction 3).* In manufactured percussion, preferred tempo should be displaced from the body-percussion baseline as a function of the mass and rotational inertia the implement adds. A heavier stick, or one whose mass is distributed further from the wrist, should lower preferred rate by an amount predictable from the change in the moment of inertia of the compound limb-plus-implement system. Neural resonance theory, dynamic attending, and predictive coding predict no such shift, because a change in the mass of an implement changes nothing in the acoustic input and nothing in a neural oscillator.

The second prediction is the more discriminating, because the first could be reconciled with rival accounts by appeal to lifelong exposure, whereas the offset prediction cannot: acoustic input and listener are held constant, and only the mechanics of the compound effector change. Body percussion thus supplies the *zero-offset baseline* against which instrumental displacement is measured, which is why it stands in the title rather than a footnote.

### 5.3 Effector stratification: the prediction and the one existing dataset

Body percussion is performed not with a single effector but with a set of them, fingers snapping, palm against palm, hands on the chest, hands on the thighs, feet on the floor, and these differ systematically in their mechanics. The relevant variable is the natural frequency of the oscillating segment, which falls broadly as segment length and effective inertia rise. A finger snap uses a short, light segment with a high natural frequency; a clap oscillates the forearm and hand about the elbow, at an intermediate frequency; a foot strike recruits the leg, the heaviest and slowest oscillator.

SEM therefore predicts a *stratification of preferred striking rate by effector*, ordered by natural frequency, fastest for fingers and hands, intermediate for chest and thigh strikes, slowest for the legs, and within a single effector class, rate scaling inversely with the length of the segment swung. This is Prediction 5.

#### 5.3.1 Preliminary evidence, and why it is unusual

To my knowledge only one kinematic study of body-percussion movement has been published (Alonso-Marco and Romero-Naranjo, 2022). It was designed to establish whether the basic movements fall within safe ranges of joint motion for older adults, and measured execution tempo only as a control variable, to standardize the speed at which each exercise was performed for video analysis. Tempo was not the object of interest, and no hypothesis about effector-dependent rate was in play. The stratification reported below is therefore an incidental regularity in data collected for an unrelated purpose, a stronger evidential position than a result from a study designed to find it.

Using two-dimensional photogrammetry and a metronome, the study recorded, for a single trained participant, the execution rate and peak segmental velocity of six basic body-percussion movements. The rates are shown in Table 1.

**Table 1:** Execution rate and peak velocity by effector in the six basic body-percussion movements, as reported by Alonso-Marco and Romero-Naranjo (2022).

Movement	Principal effector	Execution rate (bpm)	Peak velocity (m/s)
Finger snaps (chasquidos)	fingers / phalanges	90	1.47
Palms (clap)	forearm–wrist	80	1.64
Palms–thorax	arm (shoulder–elbow)	80	1.78
Palms–legs	arm (long excursion)	80	2.13
Square (cuadrado)	leg (stride 52.0 cm)	75	0.94
Triangle (triángulo)	leg (stride 63.7 cm)	65	0.99

Tempo was recorded as a control variable, not as an outcome.

#### 5.3.2 The anomaly, taken first

One feature of Table 1 is not consistent with SEM, and I take it first, because it is what a reader will notice and the honest reading of the table depends on it. The three upper-limb movements, the clap, palms-to-thorax, and palms-to-legs, were all executed at 80 beats per minute; they did not differ. Yet they recruit measurably different effectors: the clap oscillates the forearm and hand about the elbow, palms-to-thorax adds shoulder flexion, and palms-to-legs involves the largest excursion, with an elbow range of motion of 212 degrees against 109 for the clap. SEM predicts that effectors with different natural frequencies carry different rates. Within the upper limb, they did not.

Three readings are available and the data cannot distinguish them. The differences in natural frequency among these three configurations may be small relative to a metronome set in whole beats per minute, so a real difference was rounded away. The instructed-tempo procedure may have imposed a floor, the participant settling at one comfortable rate for all upper-limb movements. Or SEM may simply be wrong about within-limb variation, effector mechanics constraining rate only at the coarse grain of finger versus arm versus leg. I cannot adjudicate between these.

What follows is that the ordering in Table 1 rests on two comparisons, not five: fingers versus upper limb, and upper limb versus lower limb. The apparent smoothness of the gradient is partly an artifact of how the movements group. This is a real weakness, and any properly powered replication must measure whether within-limb differences exist at all, since a null there would substantially narrow SEM’s claim even if the coarse ordering survives.

#### 5.3.3 What the table does support

Three features remain, and none is anticipated by a substrate-general account. First, the *coarse ordering* is by effector natural frequency: fingers fastest, upper limb intermediate, lower limb slowest, spanning 90 to 65 bpm in one body in one session. Second, *within-class scaling holds where it can be tested*: the two lower-limb movements differ only in stride length, and the longer excursion (63.7 cm) was executed more slowly (65 bpm) than the shorter (52.0 cm, 75 bpm), the one within-limb difference large enough to test. Third, the *velocity data dissociate rate from effort*: the arm movements reach the highest velocities (up to 2.13 m/s) and the legs the lowest (0.94 to 0.99 m/s), so the slow lower-limb rates cannot be attributed to the legs working harder. They are slower because the oscillator is slower.

*The “it is geometry, not dynamics” objection.*

A natural objection presents itself and must be met rather than deflected. It runs: the tempo does not scale with the natural frequency of the effector; it scales with the distance the effector must travel. Finger snaps are fast because the excursion is small; the triangle is slow because the stride is long. This is geometry, not dynamics, and it has nothing to do with the mechanics SEM invokes.

The objection presupposes a separation the mechanics do not permit. Excursion and natural frequency are not independent for a limb: a limb swinging about a joint behaves approximately as a compound pendulum, whose natural frequency falls as the effective length of the swinging segment increases, because the moment of inertia grows faster than the restoring moment. A longer effective lever therefore *is* a lower natural frequency. To say the triangle is slower because the stride is longer, and to say it is slower because the oscillator has a lower natural frequency, are for a limb the same statement in two vocabularies. The objection does not identify a confound; it restates the prediction.

But this defense, though correct, is insufficient. It shows that geometry and dynamics cannot be separated *in the observational data*, so those data cannot decide between SEM and a rival account on which rate is set by some non-mechanical variable that merely correlates with limb length, such as perceived effort or learned convention. The coupling that protects the prediction from the geometric objection is exactly what prevents the kinematic data from being decisive.

What breaks the coupling is loading. Adding mass to an effector changes its moment of inertia, and so its natural frequency, while leaving the geometry of the gesture untouched: the same arm traverses the same arc to strike the same surface, so excursion, trajectory, and target are unchanged, and only the dynamics differ. A rival account on which rate tracks excursion, effort, or convention predicts no change; SEM predicts that preferred rate falls, by an amount computable from the altered inertia. This is Prediction 1, and why the interventional experiment cannot be replaced by better kinematics: the stratification of Table 1 shows the relationship is present, but only loading can show it is *mechanical*.

#### 5.3.4 Limitations of the kinematic evidence

The remaining limitations are serious. The study reports a single participant (*N* = 1), a 21-year-old woman of 1.64 m and 62 kg, filmed with one camera in two dimensions. More consequentially, the execution rates were not measured as *preferred* tempi: the participant performed at a deliberately reduced speed to facilitate video analysis, so what is recorded is the rate comfortably executed under instruction, not the rate spontaneously selected, and a comfort-versus-preference confound is live. What the data establish is not that SEM is true but that the stratification it predicts is present, ordered, and mechanically scaled in the only kinematic dataset that exists, and present in data not collected to look for it. That is a reason to run the properly powered study, which Section 6 specifies.

### 5.4 The ethnographic prediction, and what body percussion does not establish

The same logic extends to the ethnographic record at no experimental cost. The historically unrelated corporeal-rhythm traditions named in Section 5.2 converge on the same effector set, so SEM predicts a convergent effector-to-subdivision mapping across them, ordered by effector natural frequency rather than cultural transmission. The evidential force lies in the absence of a common source: a cultural account predicts no convergence across traditions that share no history, while a mechanical account predicts it, because the bodies are the same even where the cultures are not. The corpora exist; the analysis has not been done. This is part of Prediction 5.

Finally, a caution. Even if the stratification survives replication, it does not establish the mechanistic claim on its own, since a defender of the substrate-general accounts could argue that effector-to-rate assignments reflect what is *comfortable* rather than *metrically preferred*. That objection is answerable only by the interventional experiments of Section 6, where perceptual judgment, not motor output, is the dependent variable. Body percussion supplies the observational half of the program, not a substitute for the interventional half.

## 6 Falsifiable predictions and boundary conditions

A theoretical claim earns its place only if it can be wrong in a way that distinguishes it from its rivals. Each prediction below is paired with the competitor it discriminates against and with the result that would falsify it, and Table 2 summarizes them. Predictions 1 and 3 are decisive: a robust null on either would falsify the mechanistic claim.

**Table 2:** Summary of the eight predictions, the claim each tests, and the rival account each discriminates against.

#	Prediction	Claim tested	Rival prediction	Falsified if
1	Biomechanical recalibration of preferred tempo and subdivision under effector loading	Mechanistic	Substrate-general accounts predict invariance	Robust null at the perceptual level
2	Effector-specific transfer, no generalization to unloaded effectors	Mechanistic	Arousal accounts predict undifferentiated transfer	Shift transfers to unloaded effectors
3	Instrumental offset by implement mass and inertia, acoustics matched	Mechanistic	All rivals predict invariance	No displacement under matched acoustics
4	Zero offset in body percussion relative to effector natural frequencies	Mechanistic + conjectural	Rates should not differ from any other instrument	Systematic offset with no implement present
5	Effector stratification of rate and subdivision (partial preliminary support; Table 1)	Mechanistic + conjectural	Rate independent of effector	Unordered or inverted mapping; no within-limb variation
6	Orofacial extension: vocal percussion favors faster subdivisions	Mechanistic	Same parameters govern all effectors	Vocal and limb percussion share preferred subdivisions
7	Vestibular and motor dominance under cross-modal conflict	Causal	Acoustic cue dominates	Acoustic cue always wins
8	Externalization without offloading: instrument use does not degrade unaided timing	Conjectural	No prediction	Instrumentalists show degraded body-percussion timing

Predictions 1 and 3 are decisive: A robust null on either would falsify the mechanistic claim.

### 6.1 Interventional tests of the mechanistic claim

Prediction 1. Biomechanical recalibration, with a graded outcome. Altering the effective dynamics of an effector, through distal mass loading, mechanical constraint, or altered inertia, should produce predictable shifts in preferred tempo and in the most stable metrical subdivision, tracking the changed natural frequency of the effector, while the acoustic input and the listener’s musical history are held constant. As set out in Section 4.3.6, SEM predicts a graded outcome rather than a uniform effect, and the gradient is itself the prediction: the perceptual judgments are diagnostic, loaded-effector synchronization is only a manipulation check, and unloaded effectors should not shift. *Rival predictions:* neural resonance theory, dynamic attending, and predictive coding, being substrate-general, predict no shift at any level from a purely peripheral manipulation that leaves the input unchanged; ASAP predicts a shift only if the covert simulation tracks peripheral mechanics, which it does not require; an attentional-arousal account predicts an undifferentiated shift that transfers across effectors, which SEM specifically denies. *Falsification:* a robust, well-powered null at the perceptual level would falsify the mechanistic claim.

Prediction 2. Effector-specific transfer, without generalization. The metrical biases induced by movement should be specific to the oscillatory parameters of the effector moved, so that training with a slow-oscillating effector and a fast-oscillating one yields systematically different preferred subdivisions, with no transfer to unmanipulated effectors. This extends the logic of the Phillips-Silver and Trainor (2007) own-body manipulation from a category (duple versus triple) to a continuous parameter, and sharpens the observation that different effectors already carry different optimal rates (Barchet et al., 2024) into a causal test. *Rival prediction:* an account on which movement heightens general attention to the beat predicts transfer across effectors and no parameter-matched structure. The dissociation between Predictions 1 and 2 is the cleanest single discriminator SEM offers.

Prediction 3. Instrumental offset. Preferred tempo and subdivision on a manufactured percussion instrument should be displaced from the body-percussion baseline as a quantitative function of the mass and rotational inertia the implement adds, with the acoustic output held constant. A heavier implement, or one whose mass is distributed more distally, should lower preferred tempo by an amount predictable from the change in the moment of inertia of the compound limb-plus-implement system.

*A design problem that must be solved, not assumed away.* Holding the acoustic output constant across implements is harder than it sounds. A heavier stick striking with the same kinematics delivers more momentum and so produces a louder sound. Two obvious remedies fail: instructing the participant to strike more softly changes the task, not merely the plant, destroying the dissociation; accepting the louder sound leaves the input unmatched, so any tempo shift could be attributed to loudness. The design must hold the sound constant without instructing any change in movement. Three routes are available and a serious test should combine them: (i) vary the compliance of the striking surface inversely with implement mass, calibrated to yield matched sound-pressure level and spectral centroid at matched impact momentum; (ii) decouple sound from impact by striking a silent pad that triggers a fixed sample, so every strike sounds identical; (iii) take the perceptual measure while the effector is loaded but not striking, with the participant adjusting a metronome while holding the weighted implement. The third is cleanest but weakens the link to percussive practice, and is best run alongside the others. *Rival predictions:* every substrate-general account, and every task-level reading of ASAP, predicts invariance. Any displacement places transparency above zero, and its size, relative to what the altered inertia predicts, estimates how far above. This is the cheapest informative experiment SEM proposes and the cleanest available measurement of biomechanical transparency.

Prediction 4. Zero offset in body percussion. In body percussion, where effector and instrument coincide, preferred striking rates and most stable subdivisions should match the natural oscillatory frequencies of the limb segments employed, with no systematic offset, supplying the baseline against which Prediction 3 measures displacement.

### 6.2 Observational and comparative tests

Prediction 5. Effector stratification of rate and subdivision. Preferred striking rates and subdivisions in body percussion should be ordered by the natural frequencies of the effectors employed, fastest for fingers and hands, intermediate for chest and thigh strikes, slowest for the legs, and within an effector class rate should scale inversely with the length of the segment swung. Across the historically unrelated corporeal-rhythm traditions of Section 5.4, the assignment of subdivisions to effectors should converge on the same mechanically determined ordering. *Status of the evidence:* this prediction has partial preliminary support, of an unusually clean kind because it was not sought (Table 1), but the three upper-limb movements did not differ despite recruiting measurably different effectors, and the data come from one participant at instructed tempo. The properly powered study must measure preferred rather than instructed rate, across the full set of effectors, in a sample large enough to estimate the scaling exponent, and test whether loading an effector displaces its preferred rate as its altered mechanics predict. *Rival prediction:* substrate-general accounts predict that rate should not depend on which effector produces the sound; a comfort-based account predicts that any stratification should vanish when the dependent variable is a perceptual judgment rather than a motor output. *Falsification:* an unordered or inverted mapping, or a scaling exponent inconsistent with segment mechanics, would count strongly against SEM.

Prediction 6. Orofacial extension. Vocal-percussive practices, beatboxing, *konnakol*, *bol*, scat, should show preferred subdivisions matched to orofacial rather than limb natural frequencies, and should therefore favor faster subdivisions than hand or foot percussion within the same tradition, by a factor predictable from the ratio of the respective natural frequencies. This transposes to meter the argument already made for speech (Poeppel and Assaneo, 2020). *Rival prediction:* substrate-general accounts predict that the same metrical parameters should govern all effectors, since the parameters are not effector-bound.

### 6.3 Tests of the causal claim

Prediction 7. Vestibular and motor dominance under conflict. Extending the movement-determines-meter paradigm into adult, cross-modally conflicting designs, where bodily and acoustic cues to meter are pitted against one another, vestibular or motor-planning disruption should degrade metrical disambiguation more than equivalent disruption of purely auditory processing. SEM is supported only if the body-side manipulation dominates, or at least competes on equal terms, where the two conflict; if the acoustic cue always wins, the causal claim is confined to the narrow case of fully ambiguous stimuli and SEM is correspondingly weakened. The vestibular results of Trainor et al. (2009) provide a validated route to the body-side manipulation.

### 6.4 A corroborative prediction, offered as such

Prediction 8. Externalization without offloading. Skilled percussionists synchronizing to a machine-perfect external pulse should still exhibit covert motor simulation indexed to their own effector dynamics, rather than offloading timing to the device. This is candidly the weakest prediction of the set, since a null could be attributed to the insensitivity of the measure rather than the absence of simulation. It is corroborative, not decisive, and SEM should not be defended by appeal to it if the mechanistic predictions fail; resting the hypothesis on an unfalsifiable claim of hidden simulation would commit exactly the error this article has tried to avoid.

### 6.5 Boundary conditions

Three conditions bound the predictions, each established earlier: the diagnostic measure is a perceptual judgment made while the effector is loaded, since SEM predicts a gradient of malleability (Section 4.3.6); SEM concerns the envelope of metrical parameters, not the contents a culture places within it (Section 5.1); and it predicts no transfer to unloaded effectors, the cleanest discriminator against an arousal account.

## 7 Discussion

### 7.1 What the three claims jointly establish, and what they do not

The causal claim is well supported and is not mine. Movement and vestibular state can determine the metrical interpretation of an ambiguous rhythm, and galvanic stimulation of the vestibular nerve does so without any movement at all (Phillips-Silver and Trainor, 2005, 2007, 2008; Trainor et al., 2009; and, for the sensory-motor framing this inherits, Todd and Lee, 2015). The body is upstream of the felt pulse.

The mechanistic claim builds on this and reaches further, and Section 4.3 gave it the treatment it requires. Because the motor system is a hierarchy whose levels are parameterized differently, a simulation in task space predicts when a movement will occur without representing the limb that executes it, and only a simulation descending to plant space carries biomechanical parameters, the level at which force-field adaptation operates (Shadmehr and Mussa-Ivaldi, 1994) and from which optimal feedback control derives movement (Todorov and Jordan, 2002; Scott, 2004). Since ASAP does not specify which level is recruited, and a task-level reading is coherent and arguably the default (Patel and Iversen, 2014; Cannon and Patel, 2021), SEM contributes not a deduction but a quantity the existing frameworks jointly presuppose and none of them measures: the biomechanical transparency of the metrical model. Posed as a quantity rather than a dichotomy, the claim stays falsifiable, since transparency can be zero and a null under loading would establish that it is.

The ontological claim reaches furthest and rests on the least. That manufactured percussion externalizes a bodily timing capacity it does not replace is a conceptual reframing, but the reframing generates the zero-offset, instrumental-offset, and effector-stratification predictions, and those are not conceptual at all.

### 7.2 Relation to existing frameworks: specialization, not supersession

SEM does not compete with neural resonance theory, dynamic attending, predictive coding, active inference, or ASAP. It specializes them. Each permits the metrical model to be parameterized by the body; none requires it, and each survives whether or not it is. SEM converts that permission into a commitment and pays for the commitment in falsifiability. If the predictions are confirmed, the parent frameworks are not refuted but constrained, gaining a specification of where their free parameters come from. If the predictions fail, the frameworks survive untouched and SEM does not. That asymmetry is what a hypothesis is for.

### 7.3 Limitations and the principal threats

Six limitations bound the claims. The first is developed here because it has not been treated elsewhere; the remaining five recapitulate threats already set out in the sections indicated, and are gathered here so the reader can weigh them together.

The most fundamental is that SEM specifies what determines the parameters of the metrical model, but not by what channel. The candidates are not obscure, proprioceptive afference, efference copy, vestibular reafference, or some weighted combination, and Hebbian oscillator learning supplies a plausible mechanism by which such a signal would tune the network over development (Harding et al., 2025). But naming the plausible mechanism of tuning is not the same as identifying the content of the tuning signal. The predictions survive the gap, since they concern the relation between effector mechanics and metrical perception and are agnostic about the pathway, but the gap is real: SEM is, at this stage, a hypothesis about a dependency and not yet about a mechanism in the full sense.

The remaining five are, in brief: the hypothesis reports no new data and its closest existing support is correlational or comparative rather than interventional (Dahl et al., 2014; Barchet et al., 2024); the null anthropometric result of MacDougall and Moore (2005) can be read against the mechanistic claim, a tension set out in Section 4.2 and not fully resolved; the timescale objection is weakened but not dissolved, so a null on covert perceptual measures would substantially revise the hypothesis (Section 4.3.6); the effector-stratification evidence rests on two coarse comparisons from a single participant at instructed tempo (Section 5.3); and the ethnographic prediction is open to a comfort-versus-meter confound answerable only by the interventional designs of Section 6. A reader who weighted the two adverse findings more heavily than I do would be entitled to regard the mechanistic claim as already in difficulty.

## 8 Conclusion

This article asked why musical meter takes the quantitative form it does, and proposed that the answer lies in the moving body. The proposal was split into three claims, to be accepted, doubted, or rejected in parts. The body is causally upstream of the felt pulse; this is well supported. The predictive model behind beat perception may be calibrated to the oscillatory dynamics of that body, which would explain why meter has the periods and the binary defaults it has rather than any others. Section 4.3 gave this claim the treatment it requires: not a deduction from action simulation, which does not go through, but a quantity the existing frameworks jointly presuppose and none of them measures. A quantity can be measured and can come out zero; a smuggled premise can only be exposed.

Body percussion is what gives the predictions their edge, and why it stands in the title rather than a footnote. It supplies the zero-offset baseline against which an implement’s displacement is measured, and that displacement, a heavier stick lowering preferred tempo by an amount mechanics predicts with acoustics held constant, is a result no substrate-general account anticipates and any laboratory can obtain.

I have tried to report the evidence without selection, and the two findings that count against the hypothesis are set out where they arise rather than hidden. Neither is fatal, but neither has been minimized. The hypothesis should be judged by whether its distinctive predictions survive: if preferred tempo and subdivision prove invariant under biomechanical recalibration, and no instrumental offset is found, transparency is zero and SEM should be abandoned. That it can fail, cleanly and against named alternatives, is the measure of its worth.

## Data Availability

The original contributions presented in the study are included in the article/supplementary material, further inquiries can be directed to the corresponding author.
